# The effects of educational drama on students’ key competencies: a systematic review and meta-analysis

**DOI:** 10.3389/fpsyg.2026.1870089

**Published:** 2026-10-02

**Authors:** Yuan Li, Xinyuan Zhang, Gyun Heo

**Affiliations:** 1 Department of Chinese Language and Literature, Changzhi University, Changzhi, China; 2 Department of Educational Consulting, Pukyong National University, Busan, Republic of Korea; 3 Department of Fisheries Education, Pukyong National University, Busan, Republic of Korea

**Keywords:** creativity, critical thinking, educational drama, meta-analysis, systematic review

## Abstract

This study systematically evaluated the effects of educational drama interventions on students’ key competencies. A meta-analysis was conducted of randomized controlled trials and controlled clinical trials published through December 2025 and identified through searches of PubMed, Web of Science, Google Scholar, Scopus, and ProQuest Central. Standardized mean differences (SMDs) with 95% confidence intervals (CIs) were calculated using a random-effects model, and exploratory subgroup analyses were performed to examine whether participant characteristics, intervention duration, and study design might contribute to between-study heterogeneity. A total of 27 studies involving 2,239 participants were included. The findings suggest that educational drama may contribute to improvements in creativity (SMD = 1.12, 95% CI [0.68, 1.57]) and critical thinking (SMD = 0.91, 95% CI [0.16, 1.67]), with positive pooled effects also observed for communication (SMD = 2.90, 95% CI [1.59, 4.20]) and collaboration (SMD = 2.00, 95% CI [0.60, 3.39]). However, substantial heterogeneity was observed across all outcomes. Sensitivity analyses indicated that the pooled effects for communication and critical thinking remained statistically significant after the exclusion of studies reporting unusually large effect sizes, whereas the pooled effect for collaboration became non-significant after the exclusion of the study reporting the largest effect size. Overall, the available evidence suggests that educational drama may contribute to improvements in students’ creativity, critical thinking, communication, and collaboration. Nevertheless, these findings should be interpreted with caution because of the substantial heterogeneity, the influence of several studies reporting unusually large effect sizes, and the absence of formal, design-specific risk-of-bias assessments, such as RoB 2 and ROBINS-I. Future research employing more rigorous study designs and standardized risk-of-bias assessment procedures is needed to strengthen the evidence base.

**Systematic Review Registration:**

Identifier INPLASY (INPLASY202640038).

## 1 Introduction

Educational drama is an instructional approach that integrates the concepts, elements, and expressive techniques of drama and theatre into school-based teaching contexts. Its defining feature is the use of drama as an educational medium to support instructional goals and learning processes in a flexible manner, rather than treating performance outcomes as the ultimate objective.

Educational drama encompasses a range of related drama-based pedagogical traditions rather than a single, uniform instructional model. These traditions include drama in education, creative drama, process drama, theatre education, drama pedagogy, role-playing, and other drama-based approaches. Although they differ in their theoretical emphases and modes of instructional implementation, they all employ dramatic experiences as educational tools to promote learning through active participation, collaborative inquiry, and reflective engagement, rather than treating theatrical performance as an end in itself (Dunn, 2016; O’Neill, 1995). Among these traditions, process drama has been described as an inquiry-oriented genre within educational drama in which teachers and learners collaboratively explore problems, situations, and themes through improvised dramatic action without a scripted performance or an external audience (Dunn, 2016).

From a theoretical perspective, this idea can be traced back to the educational thought of Jean-Jacques Rousseau during the French Enlightenment. In Emile, Rousseau argued that children should learn primarily through experience rather than verbal instruction and advanced the principle that experience should precede formal curricular instruction (Weiler Gur Arye, 2022). Similar ideas are also reflected in John Locke’s educational philosophy, which emphasized learning through experience and observation rather than rote instruction (Locke, 1996). Together, these educational philosophies laid important foundations for the subsequent development of experiential learning and drama-based educational practices.

Building on this experiential educational philosophy, Dorothy Heathcote developed the Mantle of the Expert approach, in which drama serves as a medium for inquiry and learning rather than as an end in itself. This approach positions students as active participants who construct knowledge through dramatic roles and authentic problem-solving activities (Heathcote and Herbert, 1985). O’Neill (1995) subsequently further developed and systematized the theoretical foundations of process drama, emphasizing collaborative exploration, role enactment, and reflective meaning-making through improvised dramatic experiences (Bolton and Heathcote, 1995).

Within contemporary educational discourse, frameworks for 21st-century learning increasingly emphasize student-centered pedagogy and the development of four core competencies: communication, collaboration, critical thinking, and creativity (Kerkhoff, 2017). Recent research has further conceptualized educational drama as a contemporary pedagogical approach that integrates arts-based learning with classroom instruction. Rather than functioning solely as an artistic activity, drama-based pedagogy incorporates role-play, dramatic storytelling, collaborative inquiry, and reflective participation into everyday teaching practices, thereby creating authentic learning experiences that actively engage students in knowledge construction. Such approaches have been increasingly adopted across different subject areas because they encourage active participation, meaningful interaction, and learner-centered instruction, thereby supporting both academic learning and broader educational development (Mastrothanasis and Kladaki, 2025). From this perspective, educational drama provides not only opportunities for artistic expression but also a pedagogical framework for developing students’ key competencies. Drawing on the 5Cs framework—collaboration, communication, critical thinking, creativity, and cultural awareness—recent research suggests that collaboration and communication serve as important socio-cognitive processes through which drama activities support higher-order thinking. Through role negotiation, collaborative problem-solving, and reflective discussion, students are encouraged to analyse different perspectives, construct shared understandings, and develop critical and creative thinking concurrently (Hu and Shu, 2025). Against this backdrop, educational drama is particularly well aligned with contemporary educational priorities. Through role-playing, scenario construction, and collaborative interaction, it not only supports students’ understanding of academic content but also fosters emotional engagement, social development, and creative capacity. Its educational aims are therefore closely aligned with current competency-based approaches to teaching and learning (Mastrothanasis and Kladaki, 2025; Hu and Shu, 2025). Educational drama has consequently been increasingly recognized as a potentially valuable pedagogical approach for promoting students’ knowledge construction, skill development, and personal growth in contemporary educational practice.

In the present review, educational drama was used as an umbrella term encompassing these related drama-based pedagogical approaches. Accordingly, interventions described in the literature as drama in education, creative drama, theatre education, drama pedagogy, process drama, and other comparable drama-based approaches were considered eligible when they met the predefined inclusion criteria. Although these approaches differ in their pedagogical traditions and modes of classroom implementation, they all employ dramatic experiences to facilitate learning through participation, inquiry, and reflection. They were therefore considered conceptually comparable for the purposes of the present meta-analysis. Nevertheless, variation among these pedagogical traditions may represent a potential source of the substantial heterogeneity observed across the included studies and should be considered when interpreting the pooled findings. The specific intervention categories identified in the primary studies were therefore recorded and reported in Table 1 for descriptive purposes, whereas all eligible drama-based interventions were quantitatively synthesized under the broader conceptual framework of educational drama.

**Table 1:** Characteristics of the included studies.

Authors	Course	*n* (total)	Average age	Study design	Randomization	Comparison group	Type of outcome/s	Outcome measure	Age group	Frequency of intervention	Intervention period	Attrition	Country
Gökçearslan Çifci et al. (2017)	Creative drama	54	NA	CCT	Non-random allocation	Regular instruction / no creative drama program	Communication	Communication Skills Assessment Scale / İBDÖ	Undergraduate	Once a week	12 weeks	NR	Turkey
Momeni et al. (2017)	Creative drama	52	5 years	RCT	Individual randomization after cluster sampling	Regular preschool activities / no creative drama	Creativity	Jean-Louis Cellier Creativity Test	Preschool students	Twice a week	8 weeks	NR	Iran
Zaghloul (2018)	Self-development course using drama	140	NA	CCT	Non-random allocation	Traditional instruction	Communication / Critical thinking	Observation Checklist for communication and thinking skills	Undergraduate	NA	5 weeks	NR	Saudi Arabia
Karakelle (2009)	Creative drama	30	NA	CCT	Non-random allocation	No creative drama course	Creativity	Circle Drawing Test; Alternate Uses of Objects Test	Postgraduate	Once a week	10 weeks	NR	Turkey
Boal-Palheiros et al. (2023)	Drama education	108	6.84 years	RCT	Unclear group allocation	Passive control / regular school activities	Communication / Collaboration	Social Skills Rating System (SSRS)–Teacher Form	Primary school students	Once a week	30 weeks	NR	Portugal
Abac Karadeniz et al. (2015)	Creative drama	30	NA	RCT	Individual randomization	No creative drama training	Collaboration	Social Skills Evaluation Scale / SBDÖ	Junior middle school student	Twice a week	10 weeks	NR	Turkey
Uzun (2019)	Drama education	50	NA	RCT	Unclear randomization	Traditional visual arts instruction	Critical thinking	Critical Thinking Scale (Demir, 2006)	Primary school students	NA	5 weeks	NR	Turkey
Arslan et al. (2010)	Creative drama	48	NA	RCT	Individual randomization reported	Regular communication skills education	Communication	Communication Skills Evaluation Scale / İBDÖ	Undergraduate	Once a week	8 weeks	NR	Turkey
Çnar et al. (2019)	Creative drama	75	19.5 years	CCT	Non-random allocation	No creative drama activities	Communication / Critical thinking	Communication Skills Scale (CSS); California Critical Thinking Disposition Inventory (CCTDI)	Undergraduate	Once a week	14 weeks	NR	Turkey
Şenol and Metin (2021)	Drama education	36	5.25 years	CCT	Non-random allocation	Regular preschool education	Collaboration	Interaction Rating Scale of Children (IRSC); Collaboration–Cooperation–Sharing Behaviors Observation Form (CCSBOF)	Preschool students	Twice a week	8 weeks	NR	Turkey
Albalawi (2014)	Drama education	42	NA	RCT	Cluster randomization	Traditional English instruction	Creativity	Torrance Tests of Creative Thinking (TTCT)	Junior middle school student	Four classes a week	2 weeks	NR	Saudi Arabia
Holochwost et al. (2024)	Theater education	93	NA	CCT	Quasi-random grade-based assignment	Regular school activities / limited theatre exposure	Collaboration	Social Skills Improvement System (SSIS) Empathy Subscale; Social Ecogram; Narrative Perspective-taking Tasks	Primary school students	NA	15 weeks	NR	United States
Engin Gökbel (2019)	Creative drama and art activity	30	4.5 years	RCT	Individual randomization	Art activity only	Creativity	Test for Creative Thinking–Drawing Production (TCT-DP)	Preschool students	About 1–2 times a week	10 weeks	NR	Turkey
Celume et al. (2020)	Drama pedagogy training	126	9.5 years	RCT	Group randomization	Active control group (CSG)	Collaboration	Theory-of-Mind Tasks; Collaborative Behavior Assessment	Primary school students	Once a week	6 weeks	NR	France
Garaigordobil and Berrueco (2011)	Cooperative-Creative Play Program	86	5.5 years	RCT	Random assignment	Regular preschool curriculum	Creativity	Torrance Tests of Creative Thinking (TTCT); Creative Personality Behaviors and Traits Scale (EPC)	Preschool students	Once a week	30 weeks	NR	Spain
Dilmaç and Dilmaç (2020)	Creative drama	50	NA	CCT	Non-random allocation	Traditional teaching	Critical thinking	Critical Thinking Scale (Demir, 2006)	Primary school students	NA	5 weeks	NR	Turkey
Yasar and Aral (2012)	Drama education	80	6.35 years	RCT	Unclear randomization	Regular preschool curriculum	Creativity	Test for Creative Thinking–Drawing Production (TCT-DP)	Preschool students	Twice a week	12 weeks	NR	Turkey
Freeman et al. (2003)	Creative drama	93	NA	RCT	Random assignment; Solomon four-group design	Regular activity classes / no creative drama treatment	Collaboration	Social Skills Rating System (SSRS)	Primary school students	Once a week	18 weeks	NR	United States
Gündoan et al. (2013)	Drama education	120	11.5 years	CCT	Non-random allocation	No drama program	Creativity	Kujawski Creative Imagination Test	Primary school students	Twice a week	12 weeks	NR	Turkey
Hui and Lau, 2006	Drama education	195	NA	RCT	Random selection and group assignment	Other extracurricular activities	Communication	Story-Telling Test (STT)	Primary school students	Once a week	16 weeks	NR	Hong Kong
Hainselin et al. (2018)	Improvisational theater session	30	11.53 years	CCT	Non-random allocation	Sport practice control group	Creativity	Alternative Uses Task (AUT)	Junior middle school students	Once a week	11 weeks	5 excluded	France
Dima et al. (2020)	Drama education	50	10.5 years	CCT	Non-random allocation	Regular classroom instruction	Critical thinking	Cornell Class-Reasoning Test (Form X)	Primary school students	Once a week	12 weeks	NR	Greece
Molu and Baş et al. (2024)	Creative drama	66	21.12 years	RCT	Individual allocation reported	PowerPoint-based traditional teaching	Creativity	Marmara Creative Thinking Tendencies Scale	Undergraduate	Once a week	6 weeks	8 excluded	Turkey
Dima et al. (2020)	Theater education	400	9 years	RCT	Random selection/allocation reported	Regular teaching / no educational drama workshops	Critical thinking	Cornell Class-Reasoning Test (Form X); Starkey’s Questionnaire-based critical thinking questionnaire	Primary school students	Once a week	16 weeks	NR	Greece
Liu (2023)	Children’s comedy drama training	60	9 years	CCT	Non-random class allocation	No intervention	Creativity	Cognitive ability test; Creativity test	Primary school students	Twice a week	8 weeks	NR	Thailand
Şahin (2022)	Drama education	31	16.4 years	RCT	Individual randomization	Regular vocational education	Creativity	Modified TTCT Figural Form “Circles” Activity	Special Education Vocational Training Center	Twice a week	8 weeks	11 excluded	Turkey
Uzunöz and Demirhan (2017)	Creative drama	64	22.6 years	RCT	Individual randomization reported	Regular teaching program without drama classes	Critical thinking	California Critical Thinking Skills Test (CCTST); California Critical Thinking Disposition Inventory (CCTDI)	Undergraduate	Once a week	10 weeks	0	Turkey

Complete bibliographic information for all studies included in this Table is provided in the accompanying Appendix A.

Although many empirical studies have reported positive effects of educational drama on creativity and critical thinking, the findings have not been entirely consistent. Some studies have reported significant improvements, whereas others have found limited or non-significant effects (Burton et al., 2000; Karwowski and Soszynski, 2008; Dillard, 1982; Hetland and Winner, 2001; Dümenci, 2017; Sevinç et al., 2024). With respect to creativity, drama-based activities have been reported to stimulate students’ imagination and creative expression through role-playing, improvisation, and collaborative dramatic experiences (Burton et al., 2000; Hamann et al., 1991; Hui and Lau, 2006; Karwowski and Soszynski, 2008; Lin, 2010). However, this pattern has not been consistently observed. Some studies found no significant effects following drama-based interventions, suggesting that the effects of educational drama on creativity may be limited under certain conditions (Dillard, 1982; Hetland and Winner, 2001).

A similar inconsistency is evident in the literature on critical thinking. The American Psychological Association has defined critical thinking as a comprehensive higher-order cognitive capacity involving analysis, evaluation, and inference, as well as the interpretation of evidence and context-sensitive reasoning (Rapps, 1998). Üstündağ (1998) argued that creative drama contributes not only to the development of creativity and aesthetic ability but also to critical thinking, collaborative awareness, and social development through drama-based learning processes. Some studies have suggested that structured creative drama activities can, under certain conditions, facilitate the development of critical thinking (Spence-Campbell, 2008). For example, Dümenci’s (2017) training program suggested that incorporating drama activities designed to foster critical thinking into the curriculum may strengthen students’ multidimensional thinking skills. However, other empirical studies have indicated that the effects of drama-based teaching on critical thinking have not been consistently observed (Maynard, 1996; Sevinç et al., 2024). The evidence regarding communication and collaboration skills is similarly mixed. Some studies have supported the positive role of creative drama in enhancing these competencies (Arslan et al., 2010; Sarıkaya, 2020). By contrast, other research has suggested that when drama activities are confined to classroom settings, their contribution to oral expression and interactive communication may be less pronounced (Lee and Liu, 2022). Taken together, the current literature indicates considerable variation in the reported effects of educational drama on creativity, critical thinking, communication, and collaboration. Such variation may be related to differences in research design, measurement instruments, intervention duration, and cultural context. Therefore, the potential of drama-based pedagogy to foster these competencies warrants further examination within a more systematic and methodologically rigorous research framework. Despite the positive findings reported in some studies, considerable inconsistency remains across the empirical literature because of differences in participant characteristics, intervention designs, educational contexts, and outcome measures. Consequently, the overall effectiveness of educational drama in developing different dimensions of students’ key competencies remains insufficiently established, highlighting the need for a comprehensive quantitative synthesis of the available evidence.

Meta-analysis is a well-established quantitative approach to evidence synthesis that statistically integrates findings from studies addressing a specific research question (Moher et al., 2009). The term was introduced by Glass (1976) and the method uses statistical techniques to combine effect sizes across independent studies, thereby generating an overall estimate of the effect of an intervention on a given outcome. By synthesizing evidence across studies, meta-analysis may increase statistical power and reveal broader patterns that may not be apparent in any single study (Delahaye et al., 1991). Given the inconsistent findings reported in previous empirical research on the effects of educational drama on students’ key competencies, the present study adopts a meta-analytic approach to obtain more robust and reliable evidence. Compared with individual empirical studies, meta-analysis can mitigate some limitations associated with sample size, research setting, and experimental conditions, allowing the effects of educational drama to be evaluated from a more comprehensive perspective.

Although previous evidence syntheses have examined specific forms of drama-based instruction, their scope has remained relatively narrow. For example, a recent systematic review and meta-analysis focused specifically on the effects of Readers’ Theatre on reading skills, providing important evidence for literacy education but not addressing broader educational competencies such as creativity, critical thinking, communication, and collaboration (Mastrothanasis et al., 2023). Therefore, a comprehensive meta-analysis examining the effects of educational drama across multiple key competencies is still lacking. The present study addresses this gap by synthesizing evidence across four major competency domains and examining potential moderators, including participant characteristics, intervention duration, and study design. In addition to providing overall quantitative estimates of intervention effects, this review seeks to identify factors that may explain differences in educational outcomes across studies. In doing so, it contributes to the literature by providing a more comprehensive understanding of the effects of educational drama across competency domains and educational contexts. Furthermore, the findings may inform future research, support teachers in selecting and implementing drama-based pedagogies, and assist educational administrators and policymakers in designing competency-oriented curricula and educational practices.

Against this background, the present study aimed to systematically synthesize empirical evidence on educational drama interventions through meta-analysis and quantitatively estimate their overall effects on learners’ creativity, critical thinking, communication, and collaboration. It also examined whether variations in study characteristics, including participant type, intervention duration, and study design, might account for differences in intervention outcomes.

Accordingly, the present study addressed the following research questions:

RQ1: What are the overall effects of educational drama interventions on students’ creativity, critical thinking, communication, and collaboration?

RQ2: Does participant type moderate the effects of educational drama interventions on these four outcomes?

RQ3: Does intervention duration moderate the effects of educational drama interventions on these four outcomes?

RQ4: Does study design (RCT vs. CCT) moderate the effects of educational drama interventions on these four outcomes?

## 2 Materials and methods

This systematic review and meta-analysis was conducted in accordance with the Preferred Reporting Items for Systematic Reviews and Meta-Analyses (PRISMA) 2020 statement (Page et al., 2021). The study protocol was registered with the International Platform of Registered Systematic Review and Meta-analysis Protocols (INPLASY; registration number: INPLASY202640038).

### 2.1 Search strategy

A systematic search was conducted across four major bibliographic databases: PubMed, Web of Science, Scopus, and ProQuest Central. Google Scholar was additionally searched as a supplementary source to identify potentially relevant studies that might not have been indexed in these bibliographic databases. The searches were conducted between 2 and 10 January 2026 and covered records available from database inception through 31 December 2025. Studies were considered eligible if they met the predefined inclusion criteria and employed either a randomized controlled trial (RCT) or a controlled clinical trial (CCT) design. The search strategy combined subject headings, where available, with free-text terms related to students, educational drama, and the four target competencies. Boolean operators (AND and OR) were used to construct database-specific search strings. No language restrictions were applied. The complete search strings, search fields, and number of records retrieved from each database are presented in Supplementary Table S1 to enhance transparency and reproducibility. Conference proceedings, study protocols, guidelines, and other publications that did not meet the predefined eligibility criteria were excluded during the screening process.

### 2.2 Inclusion criteria

The scope of this study was defined using the PICOS framework: population, intervention, comparison, outcomes, and study design. The population comprised students participating in educational drama interventions, including those at the preschool, secondary, undergraduate, and postgraduate levels. The intervention consisted of educational drama activities delivered to the experimental groups, including creative drama and other drama-based learning activities. Comparison conditions included no drama-based learning, conventional instruction, or other educational approaches. The outcomes of interest were creativity, critical thinking, communication skills, and collaboration skills.

To ensure consistency in outcome classification, the four competency domains were categorized according to widely accepted conceptual definitions in the literature. For the purposes of this review, studies were classified as assessing creativity when the outcome reflected the generation of ideas that were both original and useful (Runco and Jaeger, 2012). Studies were classified as assessing critical thinking when the outcome measured purposeful analysis, evaluation, interpretation, inference, explanation, or self-regulated judgment (Facione, 1990). Communication outcomes referred to competencies involving the effective and appropriate exchange of ideas through communication knowledge, motivation, and skills (Spitzberg, 2013), Collaboration outcomes referred to the ability to engage effectively in coordinated activities to solve problems and achieve shared goals through shared understanding, mutual participation, and joint responsibility (Hesse et al., 2015). Because different validated instruments were used across the included studies, outcomes were synthesized according to the competency domain assessed rather than the specific instrument employed in each primary study. Eligible studies were required to use a controlled design, include both pre- and post-intervention assessments, and report intervention effects.

### 2.3 Exclusion criteria

The following types of studies were excluded: (1) studies that did not assess measurable outcomes or report relevant results; (2) conference papers, guidelines, and study protocols; (3) studies with incomplete or unavailable data; and (4) meta-analyses and review articles.

### 2.4 Study selection

All studies involving drama-based interventions or related programs were initially considered for screening. First, two reviewers independently examined the titles and abstracts of all retrieved records and excluded retracted articles, duplicate publications, studies with non-quantifiable outcomes, review articles, conference papers, and meta-analyses. When the title and abstract indicated that a study involved a drama-based intervention or related practice, the full text was obtained for further assessment. The two reviewers then independently assessed the full-text articles and excluded single-group intervention studies and studies with incomplete or unavailable data. The final set of eligible studies was subsequently determined. Any disagreements arising during the screening process were resolved through discussion with a third reviewer.

### 2.5 Data extraction

A predesigned data extraction form was used to organize and record information from the included studies. Data were extracted independently by two reviewers. The extracted information included: (1) author, (2) year of publication, (3) type of intervention, (4) sample size, (5) mean age, (6) study design (RCT or CCT), (7) randomization procedure, (8) comparison condition, (9) outcome type and measurement instrument, (10) participant age group, (11) intervention frequency, (12) intervention duration, (13) participant attrition, and (14) country. Because the primary studies used different validated instruments to operationalize the same competency domains, studies were grouped according to the underlying construct assessed—creativity, critical thinking, communication, or collaboration—rather than the specific measurement instrument used. The study characteristics are summarized in Table 1. The post-intervention means, standard deviations (SDs), sample sizes, and corresponding SMDs used in the meta-analysis are provided in Supplementary Table S3. After data extraction was completed, the two reviewers cross-checked the extracted information. Any discrepancies were resolved through discussion, and a third reviewer was consulted for adjudication when necessary.

### 2.6 Risk of bias assessment

The methodological quality of the included studies was independently assessed by two reviewers using the Joanna Briggs Institute (JBI) Critical Appraisal Checklist for Quasi-Experimental Studies. To maintain consistency across the included controlled intervention studies, the same checklist was applied to both randomized controlled trials (RCTs) and controlled clinical trials (CCTs). The checklist comprises nine items covering participant comparability, similarity of treatment conditions, the presence of a control group, outcome measurement, completeness of follow-up, measurement reliability, and statistical analysis. Each item was rated as Yes (Y), No (N), or Unclear (U). Any disagreements between the reviewers were resolved through discussion until consensus was reached. The detailed appraisal results are presented in Supplementary Table S2.

The JBI checklist was used to provide a general appraisal of the methodological quality of the included studies and to facilitate comparability across studies with different designs. However, it was not intended to replace design-specific risk-of-bias tools and may not fully capture sources of bias unique to randomized and non-randomized intervention studies. Formal tools specifically developed for these study designs, such as RoB 2 and ROBINS-I, were not applied in the present review. Accordingly, the assessment should be interpreted as a general methodological quality appraisal rather than a comprehensive, design-specific risk-of-bias assessment. This limitation should be considered when interpreting the findings.

### 2.7 Data analysis

The primary outcomes were the effects of educational drama interventions, including creative drama and other drama-based approaches, in the intervention groups relative to the control groups. The meta-analysis was performed using Review Manager (RevMan), version 5.4. All outcomes included in the meta-analysis were continuous variables. Effect sizes were calculated using the post-intervention means, SDs, and sample sizes reported for the intervention and control groups.

Post-intervention values were used rather than change scores because all included studies employed controlled pre–post intervention designs and post-intervention data were consistently available across studies. Baseline comparability was examined using information reported in the primary studies, when available. Many of the included studies, particularly the randomized controlled trials, reported comparable baseline characteristics or described appropriate randomization procedures. Several controlled clinical trials also reported no statistically significant baseline differences between the intervention and control groups, whereas a small number of studies either reported baseline imbalances or provided insufficient information for baseline comparisons. In several non-randomized studies, statistical methods such as analysis of covariance (ANCOVA) were used to adjust for potential baseline differences. Therefore, although post-intervention means were considered an appropriate and consistent basis for estimating intervention effects across studies, residual baseline differences could not be completely excluded, particularly in controlled clinical trials involving non-random allocation. The pooled effect sizes should therefore be interpreted with appropriate caution.

SMDs with 95% CIs were calculated as Hedges’g, which incorporates a correction for small-sample bias and is implemented in RevMan for continuous outcomes. Given the differences across studies in participant characteristics, intervention formats, and intervention durations, a random-effects model using the inverse-variance method was applied. Between-study heterogeneity was assessed using the *I*^2^ statistic and the chi-square test. When substantial heterogeneity was identified, subgroup analyses were conducted to explore its potential sources. Statistical significance was set at *p* < 0.05.

## 3 Results

### 3.1 Identification and selection of studies

A total of 1,855 records were initially identified through electronic database searches. After the removal of 414 duplicate records, 1,441 records remained for title and abstract screening. Of these, 1,343 were excluded because they did not meet the inclusion criteria. The full texts of 98 potentially relevant articles were then assessed for eligibility. Of these, 71 articles were excluded for reasons including an ineligible study design, an ineligible intervention, an unsuitable publication type, article retraction, unavailability of the full text, or insufficient quantitative data. Ultimately, 27 studies met the inclusion criteria and were included in the qualitative synthesis and meta-analysis. The study selection process is presented in Figure 1.

**Figure 1:**
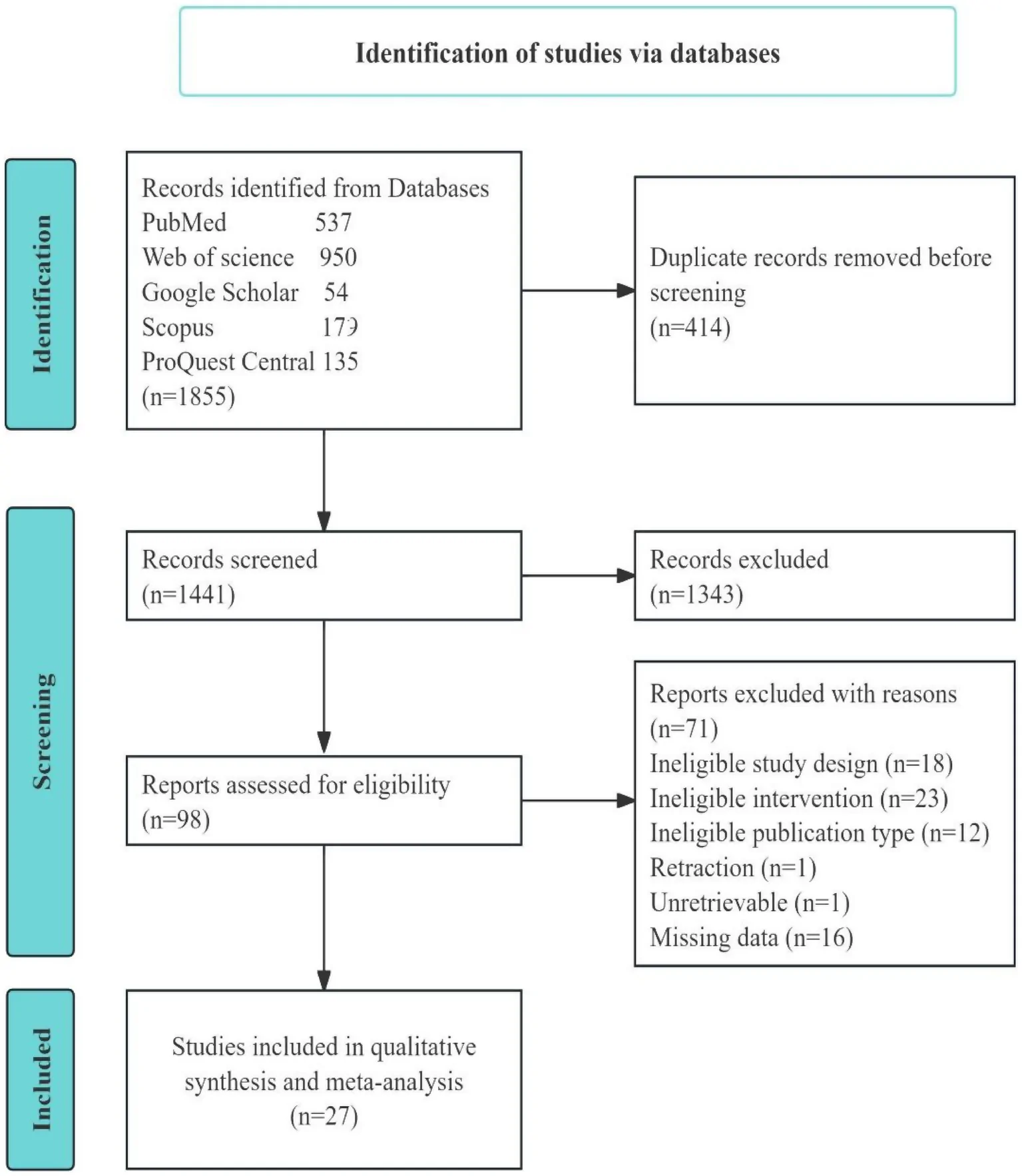
PRISMA flow diagram of the study selection process.

### 3.2 Characteristics of the included studies

A total of 27 intervention studies involving 2,239 participants were included in this review, with sample sizes ranging from 30 to 400. The geographical distribution of the studies was relatively concentrated: six were conducted in Europe, 17 in the Middle East, two in Asia, and two in the United States. The participants spanned a broad age range and included preschool children, primary and secondary school students, university students, and learners in special education settings. Most studies focused on children and adolescents, particularly those at the preschool and primary school levels, whereas only a small number involved undergraduate or postgraduate students. A few studies also examined learners in special education settings or vocational training centers.

Regarding educational level, five studies involved preschool children, 11 involved primary school students, three involved junior middle school students, six involved undergraduate students, one involved postgraduate students, and one involved learners in special education or vocational training settings. Overall, most of the available evidence was derived from preschool and compulsory education populations (19/27, 70.4%), whereas relatively few studies focused on higher education populations (7/27, 25.9%) or learners in special education and vocational training settings (1/27, 3.7%). This distribution should be considered when interpreting the generalizability of the findings across educational levels.

The included studies employed eight distinct forms of educational drama intervention, with creative drama and drama education being the most common. Other approaches included drama-based self-development courses, improvisational drama training, teacher training in drama pedagogy, and cooperative creative play programs. Despite these variations, all interventions centered on drama activities or drama-based instructional strategies, with a consistent emphasis on participatory, experiential, and collaborative learning processes. Regarding intervention intensity, most studies delivered the interventions once or twice per week, although a few used more intensive schedules. Intervention duration also varied considerably, ranging from 2 weeks to one academic year, with interventions lasting 6 to 12 weeks being the most frequently reported. The main characteristics of the included studies are presented in Table 1.

### 3.3 Quality assessment

The methodological quality of the included studies was generally satisfactory. Three studies met all nine JBI criteria (9/9), 21 studies met eight criteria (8/9), and three studies met seven criteria (7/9). Most studies clearly reported the intervention procedures, outcome measurements, and statistical analyses. However, reporting was frequently unclear with respect to participant comparability and follow-up. The detailed quality assessment results are presented in Supplementary Table S2.

### 3.4 Meta-analysis

We examined the overall effects of educational drama on creativity, critical thinking, communication, and collaboration, as well as the heterogeneity associated with these effects. The extracted post-intervention means, standard deviations, sample sizes, and corresponding standardized mean differences for all included studies are presented in Supplementary Table S3 to enhance the transparency and reproducibility of the analyses. The main results are summarized in Table 2.

**Table 2:** Overall effects of educational drama on outcomes.

Outcome	k	Sample size (experimental/control)	SMD	95% CI	*p*	I^2^ (%)	*p* for Heterogeneity
Creativity	14	337/290	1.12	[0.68, 1.57]	<0.001	83%	<0.001
Critical thinking	8	419/410	0.91	[0.16, 1.67]	0.02	95%	<0.001
Communication	7	344/276	2.90	[1.59, 4.20]	<0.001	98%	<0.001
Collaboration	6	245/241	2.00	[0.60, 3.39]	0.005	97%	<0.001

#### 3.4.1 Effect of educational drama on creativity

A total of 14 studies were included in the analysis of creativity, involving 337 participants in the intervention groups and 290 participants in the control groups. The meta-analysis indicated that educational drama had a statistically significant positive effect on creativity, with a pooled effect size of SMD = 1.12 (95% CI [0.68, 1.57]). The test for the overall effect was statistically significant (Z = 4.98, *p* < 0.00001). Substantial between-study heterogeneity was observed (χ^2^ = 77.50, df = 13, *p* < 0.00001; I^2^ = 83%), indicating that the magnitude of the intervention effect varied considerably across studies. The forest plot showing the effect of educational drama interventions on creativity is presented in Figure 2.

**Figure 2:**
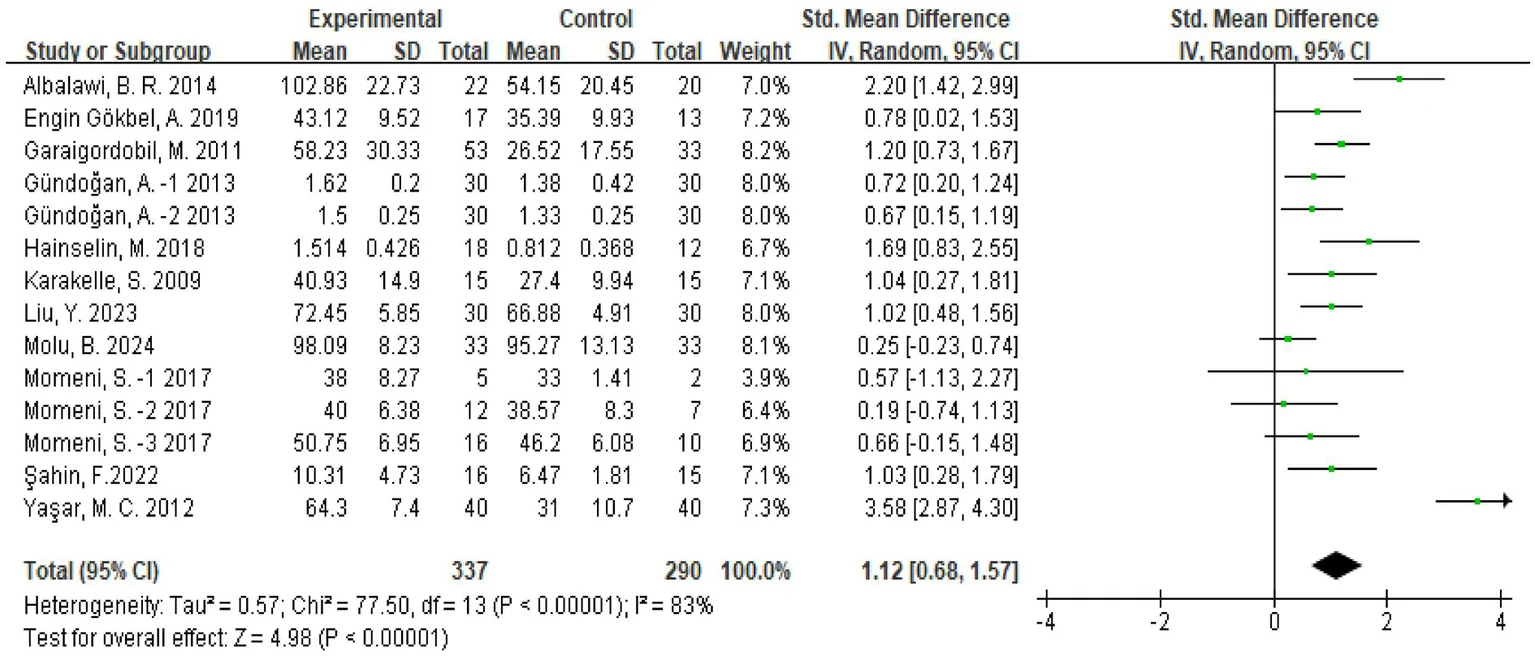
Forest plot of the effect of educational drama intervention on creativity.

#### 3.4.2 Effect of educational drama on critical thinking

Eight studies were included in the analysis of critical thinking, involving 419 participants in the intervention groups and 410 participants in the control groups. The meta-analysis showed that educational drama had a statistically significant positive effect on critical thinking, with a pooled effect size of SMD = 0.91 (95% CI [0.16, 1.67]). The overall effect was statistically significant (Z = 2.37, *p* = 0.02). Considerable between-study heterogeneity was observed (χ^2^ = 141.81, df = 7, *p* < 0.00001; I^2^ = 95%), indicating marked variation in the magnitude of the intervention effects across studies. The forest plot showing the effect of educational drama interventions on critical thinking is presented in Figure 3.

**Figure 3:**
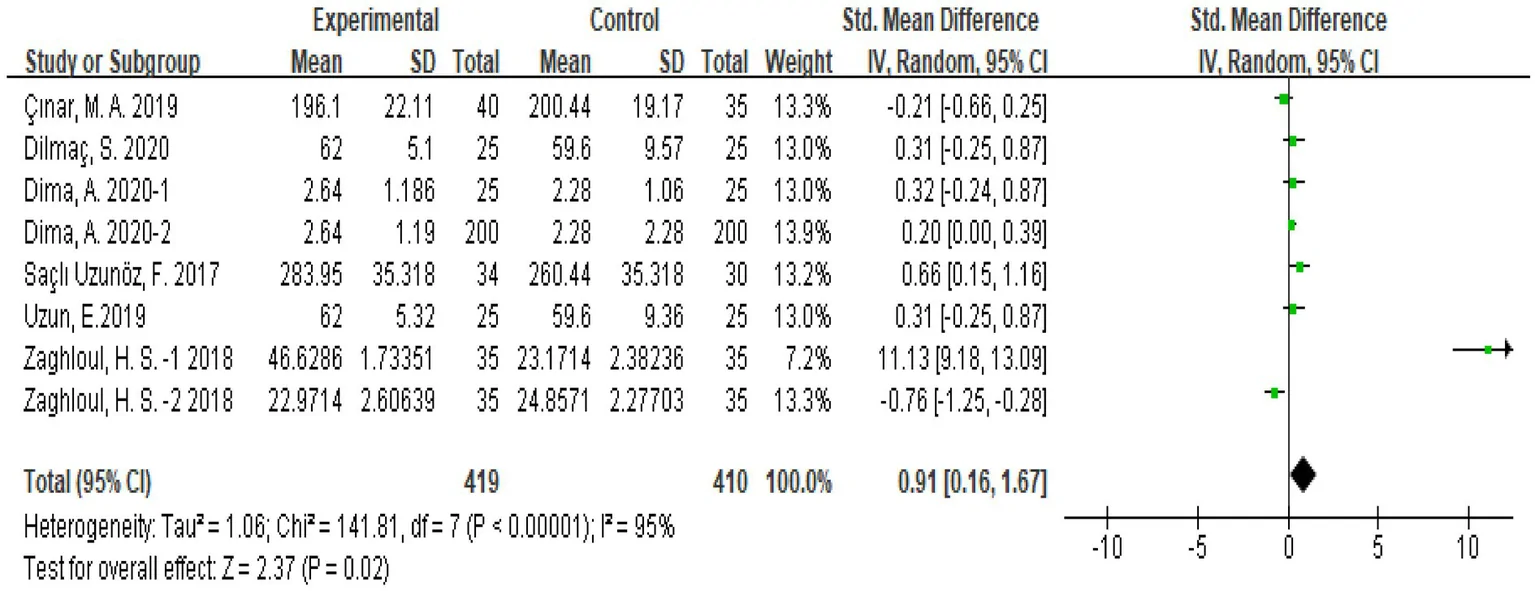
Forest plot showing the effect of educational drama intervention on critical thinking.

#### 3.4.3 Effect of educational drama on communication

Seven studies were included in the analysis of communication, involving 344 participants in the intervention groups and 276 participants in the control groups. The meta-analysis showed that educational drama had a statistically significant positive effect on communication, with a pooled effect size of SMD = 2.90 (95% CI [1.59, 4.20]). The overall effect was statistically significant (Z = 4.34, *p* < 0.0001). Substantial between-study heterogeneity was observed (χ^2^ = 246.96, df = 6, *p* < 0.00001; I^2^ = 98%), indicating considerable variation in effect sizes across the included studies. The forest plot showing the effect of educational drama interventions on communication is presented in Figure 4.

**Figure 4:**
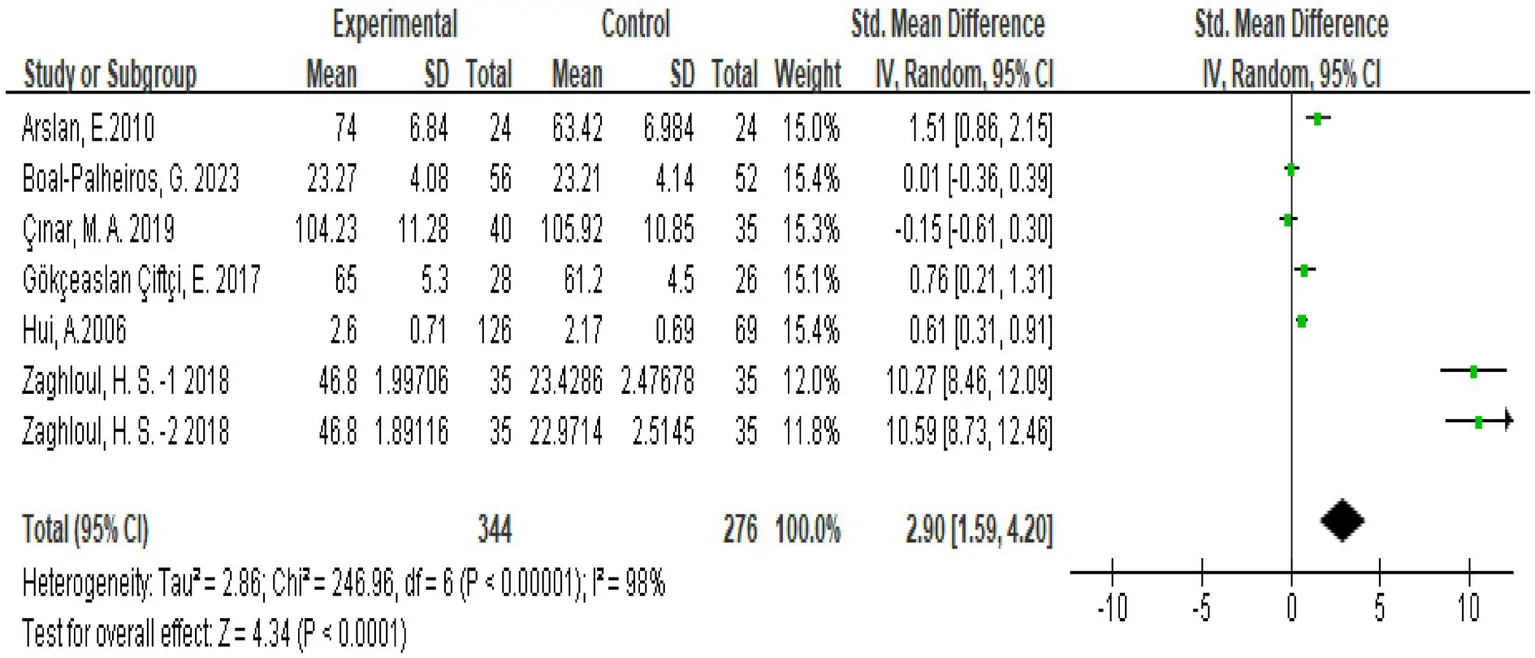
Forest plot showing the effect of educational drama intervention on communication.

#### 3.4.4 Effect of educational drama on collaboration

Six studies were included in the analysis of collaboration, involving 245 participants in the intervention groups and 241 participants in the control groups. The meta-analysis indicated that educational drama had a statistically significant positive effect on collaboration, with a pooled effect size of SMD = 2.00 (95% CI [0.60, 3.39]). The overall effect was statistically significant (Z = 2.80, *p* = 0.005). Considerable between-study heterogeneity was observed (χ^2^ = 197.76, df = 5, *p* < 0.00001; I^2^ = 97%), indicating that the magnitude of the intervention effect varied substantially across studies. The forest plot showing the effect of educational drama interventions on collaboration is presented in Figure 5.

**Figure 5:**
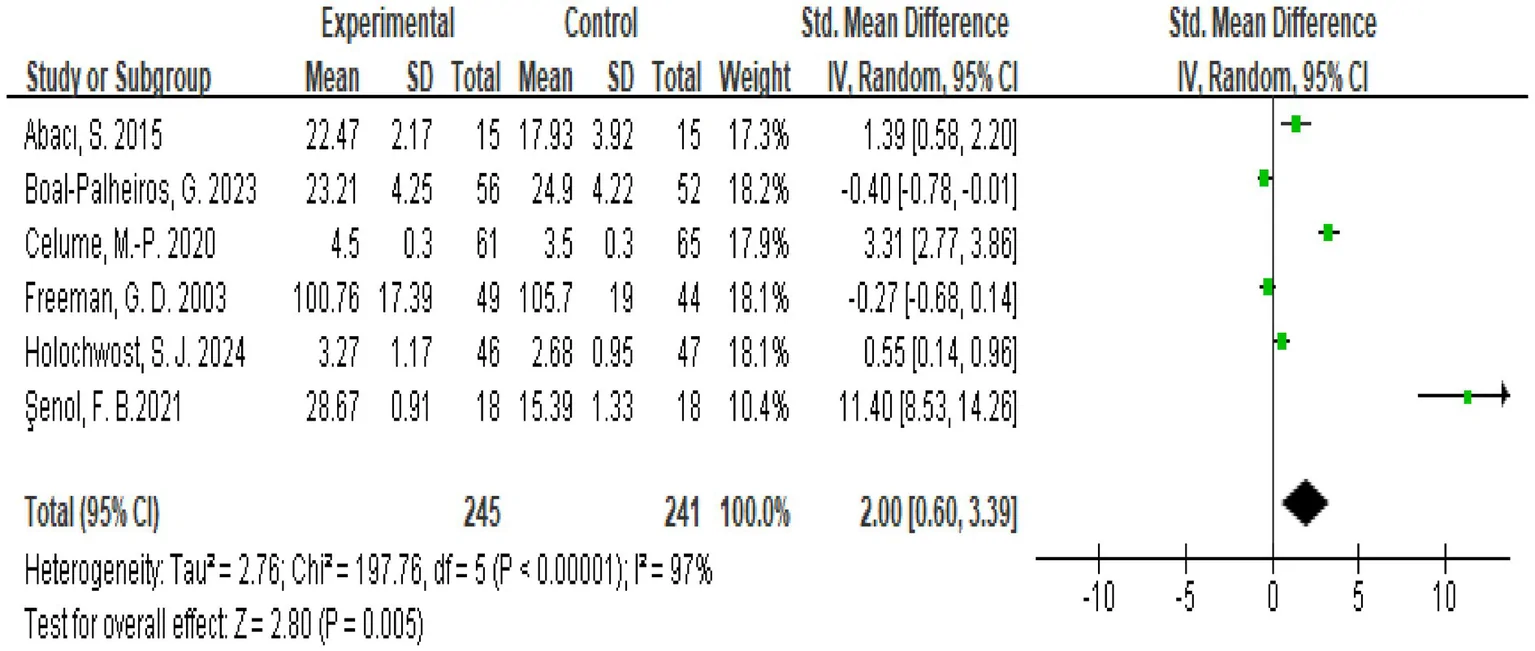
Forest plot showing the effect of educational drama intervention on collaboration.

### 3.5 Sensitivity analysis

Sensitivity analyses were conducted by excluding studies reporting extremely large effect sizes to assess the robustness of the pooled estimates. For communication, excluding the two extreme effect sizes reported by Zaghloul (2018) reduced the pooled effect size from SMD = 2.90 (95% CI [1.59, 4.20]) to SMD = 0.51 (95% CI [0.02, 0.99]), while heterogeneity decreased from I^2^ = 98% to I^2^ = 84%. The pooled effect remained statistically significant. For critical thinking, excluding the two extreme effect sizes reported by Zaghloul (2018) reduced the pooled effect size from SMD = 0.91 (95% CI [0.16, 1.67]) to SMD = 0.23 (95% CI [0.03, 0.43]), while heterogeneity decreased substantially from I^2^ = 95% to I^2^ = 24%. The pooled effect remained statistically significant.

For collaboration, excluding the study reporting the largest effect size (Şenol and Metin, 2021) reduced the pooled effect size from SMD = 2.00 (95% CI [0.60, 3.39]) to SMD = 0.90 (95% CI [−0.36, 2.17]). The pooled effect was no longer statistically significant, and heterogeneity remained high (I^2^ = 97%). These findings indicate that the pooled estimate for collaboration was more sensitive to the exclusion of an extreme effect size than those for communication and critical thinking. A summary of the original pooled estimates and the corresponding sensitivity-analysis results is presented in Table 3.

**Table 3:** Comparison of original and sensitivity-analysis pooled effect sizes.

Outcome	Original pooled effect (SMD, 95% CI)	Pooled effect after exclusion of extreme study (SMD, 95% CI)	Heterogeneity (*I*^2^)	Interpretation
Creativity	1.12 (0.68–1.57)	Not performed*	83%	No extreme effect size identified
Critical thinking	0.91 (0.16–1.67)	0.23 (0.03–0.43)	95% → 24%	Effect remained statistically significant
Communication	2.90 (1.59–4.20)	0.51 (0.02–0.99)	98% → 84%	Effect remained statistically significant
Collaboration	2.00 (0.60–3.39)	0.90 (−0.36–2.17)	97% → 97%	Effect became non-significant

Sensitivity analysis was performed only for outcomes in which studies with unusually large effect sizes were identified. No such study was identified for creativity; therefore, sensitivity analysis was not performed.

### 3.6 Subgroup analysis

The subgroup analyses revealed statistically significant between-subgroup differences for several outcomes. However, because several subgroups contained only one or two studies, these findings should be regarded as exploratory and interpreted with caution.

For creativity, significant between-subgroup differences were observed when studies were stratified by participant type (*p* for subgroup differences < 0.001), suggesting that participant characteristics may partially explain the heterogeneity observed for this outcome. Significant positive effects were found among preschool children and students at the primary and junior secondary levels, whereas the effect among undergraduate students was not statistically significant. A significant between-subgroup difference was also observed according to intervention duration (*p* for subgroup differences = 0.02). Studies involving interventions lasting ≥10 weeks yielded larger effect sizes than those involving interventions lasting <10 weeks, suggesting that intervention duration may moderate the effect of educational drama on creativity.

For communication, a significant between-subgroup difference was also observed according to participant type (*p* for subgroup differences = 0.001). A comparatively large positive effect was found among undergraduate students, whereas the effect at the primary school level was not statistically significant. A significant between-subgroup difference was also observed according to intervention duration (*p* for subgroup differences < 0.001). Interventions lasting <10 weeks yielded markedly larger effect sizes than those lasting ≥10 weeks, suggesting that intervention duration may contribute to the heterogeneity observed for this outcome.

For collaboration, significant between-subgroup differences were observed according to both participant type and intervention duration (*p* for subgroup differences < 0.001), suggesting that both factors may be associated with between-study heterogeneity. No statistically significant effect was observed for interventions lasting ≥10 weeks. Although interventions lasting <10 weeks yielded a relatively large positive effect, this effect was also not statistically significant.

Because several subgroup estimates were based on only one or a few studies, all subgroup findings should be considered hypothesis-generating rather than confirmatory. The detailed results are presented in Tables 4, 5.

**Table 4:** Subgroup analyses of the effects of educational drama on creativity and critical thinking.

Subgroup	Creativity						Critical thinking					
	Number	SMD (95%CI)	*p* (SMD)	I^2^	*p* (I^2^)	*p* (Subgroup)	Number	SMD (95%CI)	*p* (SMD)	I^2^	*p* (I^2^)	*p* (Subgroup)
Preschool students	6	1.208 (0.201, 2.215)	0.019	89.80%	<0.001	<0.001						
Primary school students	3	0.798 (0.493, 1.103)	0	0.00%	0.622		4	0.228 (0.061, 0.396)	0.008	0.00%	0.951	0.42
Junior middle school students	1	1.394 (0.585, 2.204)	0.001	—	—							
Undergraduate	1	0.254 (−0.230, 0.739)	0.304	—	—		4	2.292 (0.269, 4.315)	0.026	97.90%	< 0.001	
Special Education vocational Training Center	1	1.031 (0.275, 1.787)	0.008	—	—							
Postgraduate	1	1.039 (0.270, 1.809)	0.008	—	—							
≥10 weeks	7	1.366 (0.665, 2.066)	0	88.50%	< 0.001	0.02	4	0.22 (−0.075, 0.514)	0.144	53.00%	0.094	0.774
<10 weeks	7	0.866 (0.343, 1.389)	0.001	70.10%	0.003		4	2.392 (0.213, 4.571)	0.031	97.80%	< 0.001	
RCT	9	1.189 (0.469, 1.909)	0.001	88.80%	< 0.001	0.199	2	0.501 (0.127, 0.876)	0.009	0.00%	0.366	0.08
CCT	5	0.927 (0.631, 1.223)	0	15.10%	0.318		6	1.192 (0.180, 2.204)	0.021	96.40%	< 0.001	

Subgroup analyses were conducted according to participant type, intervention duration, and study design. Several subgroup estimates were based on only one or two studies. Therefore, these findings should be regarded as exploratory and interpreted cautiously rather than as definitive evidence of moderator effects. *p* (SMD) indicates the significance of the pooled effect size; *p* (I^2^) indicates the significance of heterogeneity; *p* (Subgroup) indicates the statistical significance of between-subgroup differences.

**Table 5:** Subgroup analyses of the effects of educational drama on communication and collaboration.

Subgroup	Communication						Collaboration					
	Number	SMD (95% CI)	*p* (SMD)	*I* ^2^	*p* (I^2^)	*p* (Subgroup)	Number	SMD (95% CI)	*p* (SMD)	*I* ^2^	*p* (*I*^2^)	*p* (Subgroup)
Preschool students							1	11.395 (8.530, 14.260)	0	—	—	<0.001
Primary school students	2	0.323 (−0.259, 0.906)	0.276	82.90%	0.016	0.001	4	0.789 (−0.668, 2.246)	0.288	97.80%	<0.001	
Junior middle school students							1	1.394 (0.585, 2.204)	0.001	—	—	
Undergraduate	5	4.373 (1.856, 6.889)	0.001	98.30%	< 0.001	0.001						
Special Education vocational Training Center												
Postgraduate												
≥10 weeks	4	0.302 (−0.117, 0.721)	0.158	76.20%	0.006	<0.001	4	0.251 (−0.404, 0.906)	0.453	87.40%	<0.001	<0.001
<10 weeks	3	7.416 (0.477, 14.355)	0.036	98.60%	<0.001		2	7.227 (−0.690, 15.143)	0.074	96.60%	<0.001	
RCT	3	0.661 (−0.022, 1.343)	0.058	87.60%	<0.001	0.053	4	0.999 (−0.707, 2.705)	0.251	97.90%	<0.001	0.275
CCT	4	5.206 (1.709, 8.704)	0.004	98.70%	<0.001		2	5.876 (−4.751, 16.503)	0.278	98.10%	<0.001	0.275

Subgroup analyses were conducted according to participant type, intervention duration, and study design. Several subgroup estimates were based on only one or two studies. Therefore, these findings should be regarded as exploratory and interpreted cautiously rather than as definitive evidence of moderator effects. *p* (SMD) indicates the significance of the pooled effect size; *p* (I2) indicates the significance of heterogeneity; *p* (Subgroup) indicates the statistical significance of between-subgroup differences.

### 3.7 Publication bias

Potential publication bias was assessed using Begg’s rank correlation test and Egger’s regression test (Egger et al., 1997). The results are presented in Table 6. For creativity, neither Begg’s test (*p* = 0.477) nor Egger’s regression test (*p* = 0.486) provided evidence of publication bias. For critical thinking, Begg’s test was statistically significant (*p* = 0.048), whereas Egger’s regression test was not (*p* = 0.192). For communication, both Begg’s test (*p* = 0.024) and Egger’s regression test (*p* = 0.008) were statistically significant, suggesting potential publication bias for this outcome. For collaboration, Begg’s test was statistically significant (*p* = 0.039), whereas Egger’s regression test was not (*p* = 0.102). Because only six studies were included in the analysis of collaboration, these findings should be interpreted with caution. Overall, the results suggest that publication bias cannot be completely ruled out, particularly for communication.

**Table 6:** Assessment of publication bias using Begg’s and Egger’s tests across outcome variables.

Outcome	Number of studies	Z	Begg’s test *p*	t	Egger’s test *p*
Creativity	14	0.71	0.477	0.72	0.486
Critical thinking	8	1.98	0.048	1.47	0.192
Communication	7	2.25	0.024	4.27	0.008
Collaboration	6	2.07	0.039	2.11	0.102

## 4 Discussion

This systematic review and meta-analysis examined the effects of educational drama interventions on students’ creativity, critical thinking, communication, and collaboration. The overall findings suggest that educational drama may have positive effects across these competency domains. Moderate to large pooled effect sizes were observed for creativity (SMD = 1.12), critical thinking (SMD = 0.91), communication (SMD = 2.90), and collaboration (SMD = 2.00). However, sensitivity analyses showed that the magnitude and robustness of these effects varied across outcomes. Excluding studies reporting unusually large effect sizes substantially reduced the pooled effects for communication and critical thinking, although both remained statistically significant. By contrast, the pooled effect for collaboration became non-significant after the study reporting the largest effect size was excluded. These findings suggest that the direction of the effects for communication and critical thinking may be relatively robust, although their original magnitudes were likely influenced by extreme estimates. The evidence for collaboration appears less stable and should therefore be interpreted with greater caution. Considerable between-study heterogeneity was observed across all outcomes (I^2^ = 83%–98%), indicating marked variation in the magnitude of the intervention effects. This variation may be associated with differences in participants’ developmental stages, intervention duration, study design, and the implementation of drama-based activities. The subgroup analyses further suggested that participant type and intervention duration may partially explain the observed heterogeneity. However, because several subgroups contained only a small number of studies, these findings should be regarded as exploratory rather than confirmatory. Overall, the findings indicate that the effects of educational drama may vary according to the educational context and the characteristics of the intervention. Taken together, the results provide preliminary evidence that educational drama may contribute to the development of students’ key competencies. Nevertheless, the certainty of the evidence remains limited by substantial heterogeneity, the influence of unusually large effect sizes, and methodological differences across studies. Although most studies met seven to nine of the nine JBI appraisal criteria, reporting was sometimes unclear regarding participant comparability and follow-up procedures. The pooled findings should therefore be interpreted cautiously.

Overall, the present findings are broadly consistent with previous evidence syntheses suggesting that drama-based pedagogies may positively influence student learning and development. For example, Mastrothanasis et al. (2023) reported that Readers’ Theatre interventions improved reading-related outcomes, highlighting the educational value of drama-based instruction in literacy contexts (Mastrothanasis et al., 2023). The present review extends this evidence by suggesting that educational drama may also contribute to broader competencies, including creativity, critical thinking, communication, and collaboration. At the same time, the substantial heterogeneity across the included studies indicates that these effects are not uniform and may vary according to participant characteristics, intervention design, educational context, and outcome measurement. Thus, the present review supports the educational potential of drama-based pedagogies while providing a broader quantitative synthesis across multiple competency domains and identifying factors that may contribute to variation in intervention effects.

With respect to students’ key competencies, the findings indicate significant positive pooled effects of educational drama on creativity and critical thinking. Educational drama may foster creativity by engaging learners in role-play, scenario construction, and improvisation. These activities place students in open-ended learning contexts in which they are encouraged to imagine, experiment with, and articulate ideas, thereby supporting divergent thinking and creative problem-solving. Compared with conventional instructional approaches centered primarily on knowledge transmission, educational drama provides a more flexible and interactive environment in which students can explore multiple perspectives and possible responses to changing situations. Its experiential nature may also provide repeated opportunities for situated practice, allowing students to refine their creative thinking through continued participation and expression. These findings are generally consistent with previous empirical evidence suggesting that educational drama can enhance imagination, divergent thinking, and creative expression (Karakelle, 2009). However, unlike individual studies reporting limited or non-significant effects (Dillard, 1982; Hetland and Winner, 2001), the present meta-analysis identified an overall positive effect by synthesizing evidence across different educational settings and intervention contexts.

A positive pooled effect was also observed for critical thinking, although the substantial heterogeneity indicates that its magnitude varied considerably across studies. This finding is generally consistent with previous research suggesting that educational drama may foster higher-order thinking through inquiry, reflection, and collaborative dialogue (Üstündağ, 1998; Spence-Campbell, 2008). However, more recent studies have reported inconsistent findings, with positive effects not always being observed across educational contexts (Sevinç et al., 2024). This inconsistency suggests that the effects of educational drama on critical thinking may depend on intervention design, implementation quality, educational context, and outcome measurement.

One plausible explanation is that educational drama creates situations involving conflict, uncertainty, and multiple perspectives, requiring learners to make judgments, consider alternatives, and reach decisions through role-based interaction. The dramatic process may therefore provide a meaningful context for the development of critical thinking. Within such scenarios, students interpret issues from different role positions and develop reasoned views through discussion and exchange. This perspective-taking may strengthen their capacities for analysis and evaluation. In addition, the discussion and reflection embedded in drama activities provide opportunities for students to articulate arguments and justify their positions, thereby further supporting critical thinking. Previous evidence suggests that contextualized learning and interactive discussion can promote the development of higher-order cognitive skills (Hmelo-Silver, 2004). Taken together, these findings suggest that educational drama may contribute to improvements in students’ creativity and critical thinking. However, differences in the instruments used to assess the same competency domains, together with variations in methodological quality, intervention characteristics, and educational contexts, may partly explain the heterogeneity observed across studies. Creativity, critical thinking, communication, and collaboration were assessed using different validated instruments across the included studies, and differences in their conceptualization and measurement may have contributed to variability in the estimated effect sizes.

Regarding students’ social interaction competencies, the present findings indicate that educational drama has significant positive effects on both communication and collaboration, although substantial heterogeneity was again observed across studies. With respect to communication, educational drama is an interactive form of learning that requires students to engage in verbal expression, emotional exchange, and meaning-making through role-play and situational interaction. These processes may provide opportunities for the development of communicative competence. Compared with conventional teacher-centered instruction, educational drama places greater emphasis on peer interaction and shared participation, enabling learners to express their ideas and respond to others in authentic or simulated contexts. It may therefore strengthen verbal expression and broader interpersonal communication skills. Drama activities also commonly involve group discussion, situational negotiation, and role-based expression, which may help students understand others’ perspectives and develop more adaptive communication strategies. Recent research on drama pedagogy further suggests that drama-based processes may support communicative development by promoting vocal awareness, attentive listening, communicative presence, and responsiveness during live interaction (Mastrothanasis, 2026). These processes provide a plausible mechanism through which educational drama may facilitate students’ interpersonal communication skills.

The present findings are consistent with previous studies suggesting that drama-based learning may enhance students’ communication skills by creating authentic opportunities for dialogue, role negotiation, and collaborative interaction (Arslan et al., 2010; Sarıkaya, 2020). Nevertheless, some studies have reported only limited improvements in oral communication when drama activities were implemented in relatively constrained classroom contexts (Lee and Liu, 2022). These inconsistent findings further suggest that the effects of educational drama may vary according to the instructional context and the quality of implementation.

A similarly positive pooled effect was observed for collaboration. Educational drama is commonly organized around group-based activities in which students negotiate roles, coordinate tasks, and co-construct shared scenarios. Such processes may provide a foundation for the development of collaborative competence. Within dramatic contexts, learners are required not only to articulate their own ideas but also to reconcile different viewpoints and work together to complete collective tasks. These features of drama-based learning may support the development of teamwork and cooperative problem-solving skills. Educational drama may also encourage students to build trust through shared activities, thereby facilitating more effective collaboration during problem solving. The present findings are broadly consistent with previous research suggesting that drama-based collaborative activities may promote teamwork, shared responsibility, and cooperative problem solving (Şenol and Metin, 2021). However, the magnitude of the pooled effect varied considerably across studies, and the sensitivity analysis indicated that the collaboration finding was less stable than those for communication and critical thinking. Differences in participant characteristics, comparison conditions, educational settings, intervention intensity, implementation, and assessment methods may have contributed to this variability. Taken together, the findings suggest that educational drama may support the development of communication and collaboration, although the magnitude and consistency of these effects appear to depend on the context and design of the intervention. Further research is needed to clarify the mechanisms and conditions underlying these potential benefits.

Sensitivity analyses provided further insight into the stability of the pooled findings. After the exclusion of unusually large effect sizes, the pooled effects for communication and critical thinking were substantially reduced but remained statistically significant, whereas the pooled effect for collaboration became non-significant after the study reporting the largest effect size was excluded. These findings suggest that the direction of the effects for communication and critical thinking was relatively stable, although their original magnitudes were influenced by extreme estimates. By contrast, the evidence for collaboration was less robust and should be interpreted more cautiously. In addition, both Begg’s and Egger’s tests indicated potential publication bias for communication. Taken together, these findings suggest that several pooled estimates may have been affected by unusually large effect sizes and selective publication. Therefore, greater caution is warranted when interpreting the magnitude of the observed intervention effects, particularly for communication and collaboration.

To further explore potential sources of between-study heterogeneity, subgroup analyses were conducted according to participant characteristics, intervention duration, and study design. The overall pattern suggests that these study characteristics may have influenced the effects of educational drama, although their associations were not consistent across outcomes. This variation indicates that the effects of educational drama may be partly context dependent. With respect to participant characteristics, the subgroup analyses suggested that the effects of educational drama varied across educational levels. For creativity, significant positive effects were observed among preschool children and students in compulsory education, whereas the effect among undergraduate students was not statistically significant. One possible explanation is that role-play, scenario construction, and improvisation may be particularly well suited to supporting imaginative engagement and divergent thinking among younger learners. By contrast, creativity in higher education may be more closely related to discipline-specific knowledge and engagement with complex problems, which may affect the extent to which students benefit from drama-based activities. For critical thinking, significant positive effects were observed among both primary school students and undergraduates, although the effect size was larger and heterogeneity was greater in the undergraduate subgroup. Students in higher education may have more developed capacities for abstract reasoning and reflective judgment, potentially enabling them to engage more fully with the interpretive and dialogic demands of drama-based learning. For communication, a relatively large effect was observed among undergraduate students, whereas the effect among primary school students was not statistically significant. This difference may be related to variations in linguistic abilities, social experience, intervention characteristics, or outcome measurement across educational levels. A similar variation was observed for collaboration, with relatively large effects in some subgroups but no statistically significant effect among primary school students. However, because several participant subgroups contained only one or a few studies, these patterns should be interpreted as exploratory rather than as firm evidence of developmental differences.

Subgroup analyses according to intervention duration also revealed variation across outcomes. For creativity, longer interventions (≥10 weeks) were associated with larger positive effects, suggesting that the development of creativity may benefit from sustained engagement and repeated practice. By contrast, shorter interventions (<10 weeks) yielded relatively large effects for communication and collaboration. This pattern may reflect the capacity of drama-based activities to promote interaction and participation within a relatively short period. However, the pooled effects for collaboration were not statistically significant in either duration subgroup and should therefore be interpreted with caution. Moreover, because several duration subgroups included only one or a few studies, the apparent differences between shorter and longer interventions should be considered exploratory.

Some variation was also observed across study designs. AlthoughCCTs tended to report larger effect estimates for several outcomes, none of the between-subgroup comparisons according to study design was statistically significant (*p* > 0.05). Therefore, the available evidence does not indicate that study design was a significant moderator of the intervention effects. The descriptive differences between RCTs and CCTs may reflect variation in participant characteristics, educational settings, intervention implementation, or methodological rigor rather than study design alone. In addition, the small number of studies in several subgroups may have reduced statistical power and limited the ability to detect between-subgroup differences. Future studies should use more standardized outcome measures, comparable control conditions, and rigorous methodological procedures to facilitate more reliable comparisons across educational contexts and forms of drama-based intervention.

Despite the systematic synthesis provided by this meta-analysis, several limitations should be acknowledged. First, experimental research on educational drama remains relatively limited, partly because such interventions can be difficult to design and implement in real-world educational settings. Studies that simultaneously assessed creativity, critical thinking, communication, and collaboration, included an appropriate control group, and reported sufficient data for effect-size calculation were particularly scarce. Consequently, the number of eligible studies was limited, which may have reduced the robustness and generalizability of the findings. Second, educational drama interventions are inherently context sensitive. Their implementation often requires considerable facilitator involvement and careful situational design, making the observed effects susceptible to contextual variation. Differences in cultural background, intervention duration, participant age and developmental stage, and implementation procedures may have contributed to the substantial heterogeneity across studies and complicated the interpretation of the pooled effects.

Third, although the included studies met between seven and nine of the nine JBI appraisal criteria, several provided incomplete information regarding participant comparability and follow-up. In addition, the use of a general JBI appraisal checklist rather than design-specific risk-of-bias tools limited the extent to which sources of bias unique to randomized and non-randomized studies could be evaluated. Substantial heterogeneity also remained across several outcomes, and sensitivity analyses indicated that some pooled estimates, particularly for collaboration, were influenced by studies reporting unusually large effect sizes. Finally, potential publication bias could not be ruled out, particularly for communication. The pooled findings should therefore be interpreted cautiously, and additional rigorously designed studies are needed to strengthen the evidence base.

In addition, although the JBI Critical Appraisal Checklist was used to provide a general appraisal of methodological quality, formal design-specific risk-of-bias tools, such as RoB 2 for randomized trials and ROBINS-I for non-randomized studies, were not applied. The present appraisal should therefore not be interpreted as a comprehensive, design-specific risk-of-bias assessment. Several subgroup analyses were also based on relatively few studies and should be regarded as exploratory. Moreover, because some studies contributed effect sizes to more than one outcome domain, complete statistical independence across all pooled analyses cannot be assumed. Finally, the classification of studies as RCTs or CCTs was based on information reported in the original publications, and incomplete reporting may have limited the accuracy of this classification.

Compared with previous reviews focusing on a single educational outcome or a specific form of drama-based instruction, the present review synthesized evidence across four key competency domains and explored potential moderators related to participant characteristics, intervention duration, and study design. This broader perspective contributes to a more comprehensive understanding of the potential educational value of educational drama across different contexts and may inform future research, educational practice, and curriculum development.

In light of these limitations, future research should place greater emphasis on rigorous intervention design, transparent reporting, and the use of standardized, design-specific risk-of-bias assessment tools in evidence synthesis. From the perspective of educational practice, the present findings suggest that educational drama may contribute to the development of students’ creativity, critical thinking, communication, and collaboration. However, given the substantial heterogeneity and methodological limitations of the available evidence, its implementation should be carefully aligned with specific curricular objectives, learner characteristics, and educational contexts.

Educational administrators and teachers may consider piloting drama-based approaches in appropriate settings, supported by adequate teacher preparation and systematic evaluation. Strategies such as role-play, scenario-based activities, and collaborative inquiry may help create more interactive learning environments and support students’ engagement, participation, and expressive abilities. Future studies should include larger and more diverse samples and conduct long-term, multi-regional investigations across different educational stages. The integration of quantitative and qualitative methods may also help clarify the mechanisms, implementation conditions, and longer-term effects of educational drama, thereby strengthening the evidence base for its use in educational practice.

## 5 Conclusion

This meta-analysis systematically synthesized the available empirical evidence on educational drama interventions and examined their effects on students’ key competencies. The findings suggest that educational drama may contribute to the development of creativity, critical thinking, communication, and collaboration across a range of educational settings. Through activities such as role-play, scenario-based learning, and collaborative interaction, educational drama may provide opportunities for students to engage in expression, discussion, perspective-taking, and problem solving within authentic or simulated social contexts.

Overall, educational drama appears to be a potentially valuable pedagogical approach for supporting students’ cognitive and social competencies. However, the findings should be interpreted cautiously because of the substantial between-study heterogeneity, the influence of unusually large effect sizes, potential publication bias for some outcomes, and limitations in the methodological assessment of the included studies. Future research should prioritize rigorously designed randomized controlled trials, standardized outcome measures, transparent reporting, and design-specific risk-of-bias assessments. Studies combining quantitative and qualitative methods may also help clarify how the effects of educational drama vary across age groups, subject areas, intervention characteristics, and cultural contexts.

## Data Availability

The original contributions presented in the study are included in the article/Supplementary material, further inquiries can be directed to the corresponding author.
