# Supplementary material for: The effects of educational drama on students’ key competencies: a systematic review and meta-analysis
Source: Front Psychol. 2026 Oct 2;17:1870089. doi: 10.3389/fpsyg.2026.1870089 (PMC13672507; doi:10.3389/fpsyg.2026.1870089)
Supplement: Supplementary file 1 [file Data_Sheet_1.PDF]

Appendix A. Complete Bibliographic Information for All Studies Included in the Meta-analysis

Abacı Karadeniz, S., Tepeli, K., and Erbay, F. (2015). Yaratıcı Drama Eğitiminin Ergenlerin Sosyal Becerilerine Etkisi. *Yaratıcı Drama Dergisi* 10. doi: 10.21612/yader.2015.016

Albalawi, B. R. (2014). Effectiveness of teaching English subject using drama on the development of students' creative thinking. *IOSR Journal of Research & Method in Education* 4, 54-63. doi: 10.9790/7388-04615463

Arslan, E., Erbay, F., and Saygın, Y. (2010). Yaratıcı Drama İle Bütünleştirilmiş İletişim Becerileri Eğitiminin Çocuk Gelişimi ve Eğitimi Bölümü Öğrencilerinin İletişim Becerilerine Etkisinin İncelenmesi. *Selçuk Üniversitesi Sosyal Bilimler Enstitüsü Dergisi*, 23, 1-8. https://izlik.org/JA72JZ78NA

Boal-Palheiros, G., and Ilari, B. (2023). Music, drama, and social development in portuguese children. *Front. Psychol.* 14:1093832. doi: 10.3389/fpsyg.2023.1093832

Celume, M., Goldstein, T., Besançon, M., and Zenasni, F. (2020). Developing children's socio-emotional competencies through drama pedagogy training: An experimental study on theory of mind and collaborative behavior. *Eur. J. Psychol.* 16, 707-726. doi: 10.5964/ejop.v16i4.2054

Dilmaç, S., and Dilmaç, O. (2020). Görsel sanatlar dersinde yaratıcı drama yönteminin öğrencilerin eleştirel düşünme becerilerine etkisi. *Yaratıcı Drama Dergisi* 15, 85-100. doi: 10.21612/yader.2020.005

Dima, A., Kaiafa, E., and Tsiaras, A. (2020). Evaluation of the educational drama as an innovative method to be adopted by teachers in order to enhance critical thinking skills of students in primary school. *Open Journal for Educational Research* . 4. doi: 10.32591/coas.ojer.0402.03103d

Dima, A., and Tsiaras, A. (2020). The improvement of critical thinking through Drama Education for students in the fifth grade of Primary school. *Drama Res*. 11(1):Article 7.

Gökçearslan Çifci E., and Altınova, H. H. (2017). Development of comunnication skills through creative drama at social work education: An example of an implementation of the course. *Elementary Education Online* 16(4), 1384-1394. doi: 10.17051/ilkonline.2017.342961

Engin Gökbel, A. (2019). *The Effect of Integrated Creative Drama-Art Activities Applied to 4–5 Years Old Pre-school Children on Creative Thinking Skills* (Master's thesis). Ankara: Ankara University. doi: 10.13140/RG.2.2.18263.59048.

Freeman, G. D., Sullivan, K., and Fulton, C. R. (2003). Effects of creative drama on self-concept, social skills, and problem behavior. *The Journal of Educational Research*. 96(3):131–138. doi: 10.1080/00220670309598801

Garaigordobil, M., and Berrueco, L. (2011). Effects of a play program on creative thinking of preschool children. *Span. J. Psychol.* 14, 608-618. doi: 10.5209/rev_sjop.2011.v14.n2.9

Gündoğan, A., Ari, M., and Gönen, M. (2013). The effect of drama on the creative imagination of children in different age groups. *Hacettepe Univ. J. Educ.* 28(2):206–220.

Hainselin, M., Aubry, A., and Bourdin, B. (2018). Improving teenagers' divergent thinking with improvisational theater. *Front. Psychol.* 9, 1759. doi: 10.3389/fpsyg.2018.01759

Hui, A., and Lau, S. (2006). Drama education: A touch of the creative mind and communicative-expressive ability of elementary school children in Hong Kong. *Think. Skills Creat.* 1, 34-40. doi: 10.1016/j.tsc.2005.06.001

Holochwost, S. J., Goldstein, T. R., and Wolf, D. P. (2024). More light about each other: Theater

education as a context for developing social awareness and relationship skills. *Psychology of Aesthetics, Creativity, and the Arts*, 18(4), 666–677. doi: 10.1037/aca0000479

Karakelle, S. (2009). Enhancing fluent and flexible thinking through the creative drama process. *Think. Skills Creat.* 4, 124-129. doi: 10.1016/j.tsc.2009.05.002

Liu, Y. (2023). The effects of children's drama on children's cognitive ability and creativity. *Journal of Multidisciplinary in Humanities and Social Sciences.* 6, 1949-1965.

Molu, B., and Baş, M. T. (2024). The impact of implementing the creative drama technique on creative thinking and empathy levels of nursing students in pediatric nursing course. *Teach. Learn. Nurs.* 19, e455-e461. doi: 10.1016/j.teln.2024.01.013

Momeni, S., Khaki, M., and Amini, R. (2017). The role of creative drama in improving the creativity of 4-6 years old children. *Journal of History Culture and Art Research.* 6, 617-626. doi: 10.7596/taksad.v6i1.765

Çınar, M. A., Dinler, E., Bayramlar, K., and Yakut, Y. (2019). The effect of creative drama on empathic tendencies, communication skills and critical thinking of physiotherapy students. *CBU Int. Conf. Proc.* 7, 711–716. doi: 10.12955/cbup.v7.1443

Şahin, F. (2022). A study on developing creative thinking skills in students with intellectual disabilities using creative drama. *Creat. Res. J.* 34, 85-92. doi: 10.1080/10400419.2021.1997177

Şenol, F., and Metin, E. (2021). Examining the effectiveness of drama education program on the interaction and social skills of children in preschool classes applying inclusive practices. *Education and Science.* 46, 189-212. doi: 10.15390/EB.2021.9033

Uzunöz, F. S., and Demirhan, G. (2017). The effect of creative drama on critical thinking in preservice physical education teachers. *Think. Skills Creat.* 24, 164-174. doi: 10.1016/j.tsc.2017.02.018

Uzun E (2019). *Ortaokul 5. sınıf görsel sanatlar dersinde yaratıcı drama yönteminin öğrencilerin eleştirel düşünme becerilerine etkisi* (Master's thesis). Erzurum: Atatürk University. Available online at: https://tez.yok.gov.tr/UlusalTezMerkezi/

Yasar, M. C., and Aral, N. (2012). Drama education on the creative thinking skills of 61-72 months old pre-school children. *Procedia Soc. Behav. Sci*. 51, 568–572. doi: 10.1016/j.sbspro.2012.08.211

Zaghloul, H. S. (2018). Using creative educational drama to enhance self-development skills for the students at university level. *Int. J. Adv. Comput. Sci. Appl.* 9(8), 81–86. doi: 10.14569/IJACSA.2018.090812.
