# Supplementary material for: The effects of educational drama on students’ key competencies: a systematic review and meta-analysis
Source: Front Psychol. 2026 Oct 2;17:1870089. doi: 10.3389/fpsyg.2026.1870089 (PMC13672507; doi:10.3389/fpsyg.2026.1870089)
Supplement: Supplementary file 2 [file Table_1.DOCX]

**Supplementary Table S1**

Complete search strategies for all electronic databases

| 1.PubMed (n = 537) | | |
| --- | --- | --- |
| # | Search strategy | Records retrieved |
| #1 | Students[MeSH Terms] | 193534 |
| #2 | (((((Students) OR (Student)) OR (School Enrollmen)) OR (Enrollment School)) OR (Enrollments School)) OR (School Enrollments) | 758312 |
| #3 | #1 OR #2 | 758312 |
| #4 | (((Thinking[MeSH Terms]) OR (communication[MeSH Terms])) OR (collaboration[MeSH Terms])) OR (creativity[MeSH Terms]) | 710344 |
| #5 | ((((((((((((((((((((((((((((((((((((((((((((((((Thinking) OR (communication)) OR (collaboration)) OR (creativity)) OR (Critical Thinking)) OR (Thinking, Critical)) OR (Thinking Skills)) OR (Thinking Skill)) OR (Thought)) OR (Thoughts)) OR (Communication Programs)) OR (Communication Program)) OR (Program, Communication)) OR (Programs, Communication)) OR (Communications Personnel)) OR (Personnel, Communications)) OR (Personal Communication)) OR (Communication, Personal)) OR (Social Communication)) OR (Communication, Social)) OR (Communications, Social)) OR (Social Communications)) OR (Miscommunication)) OR (Miscommunications)) OR (Misinformation)) OR (Misinformations)) OR (Communication Disorder, Social)) OR (Communication Disorders, Social)) OR (Social Communication Disorders)) OR (Pragmatic Communication Disorder)) OR (Communication Disorder, Pragmatic)) OR (Communication Disorders, Pragmatic)) OR (Disorders, Pragmatic Communication)) ) OR (Interpersonal Relation)) OR (Relation, Interpersonal)) OR (Social Relationships)) OR (Relationship, Social)) OR (Social Relationship)) OR (Creative Thinking)) OR (Thinking, Creative Pragmatic Communication Disorders)) OR (Creativeness)) OR (Creative Ability)) OR (Ability, Creative)) OR (Creative Abilities)) OR (Artistic Creativity)) OR (Creativity, Artistic)) OR (Originality)) OR (Innovativeness) | 3506257 |
| #6 | (#4) OR (#5) | 3506257 |
| #7 | ((((((((((((((Drama Education) OR (Theatre Education)) OR (Drama in Education)) OR (Educational Drama)) OR (drama pedagogy)) OR (Drama-based Pedagogy)) OR (drama-based learning)) OR (process drama)) OR (creative drama)) OR (applied drama)) OR (drama-based instruction)) | 6570 |
| #8 | ((#3) AND (#6)) AND (#7) | 537 |
| Records retrieved:537 | | |
| 2.Web of Science (n = 950) | | |
| # | Search strategy | Records retrieved |
| 1 | Students (All Fields) or Student (All Fields) or School Enrollment (All Fields) or Enrollment, School (All Fields) or Enrollments, School (All Fields) or School Enrollments (All Fields) | 1250633 |
| 2 | Drama Education (All Fields) or Theatre Education (All Fields) or Drama in Education (All Fields) or Educational Drama (All Fields) or drama pedagogy (All Fields) or Drama-based Pedagogy (All Fields) or drama-based learning (All Fields) or process drama (All Fields) or creative drama (All Fields) or applied drama (All Fields) or drama-based instruction (All Fields) | 10364 |
| 3 | ALL=(Thinking) OR ALL=(communication) OR ALL=( collaboration) OR ALL=( creativity) OR ALL=(Critical Thinking) OR ALL=(Thinking, Critical) OR ALL=(Thinking Skills) OR ALL=(Thinking Skill) OR ALL=(Thought) OR ALL=(Thoughts) OR ALL=(Communication Programs) OR ALL=(Communication Program) OR ALL=(Communications Personnel) OR ALL=(Personal Communication) OR ALL=(Social Communication) OR ALL=(Social Communications) OR ALL=(Social Communication Disorders) OR ALL=(Pragmatic Communication Disorder) OR ALL=(Interpersonal Relation) OR ALL=(Social Relationships) OR ALL=(Social Relationship) OR ALL=(Creative Thinking) OR ALL=(Thinking, Creative Pragmatic Communication Disorders) OR ALL=(Creativeness) OR ALL=(Creative Ability) OR ALL=(Creative Abilities) OR ALL=(Ability, Creative) OR ALL=(Artistic Creativity) OR ALL=(Creativity, Artistic) OR ALL=(Originality) OR ALL=(Innovativeness) | 3440562 |
| 4 | #1 AND #2 AND #3 | 950 |
| Records retrieved:950 | | |
| 3.Google Scholar (n = 54) | | |
| Search strategy | | |
| Students AND ("Drama Education" OR "Creative Drama") AND (Creativity OR Critical Thinking OR Communication OR Collaboration) | | |
| Records retrieved:54 | | |
| 4.Scopus (n = 179) | | |
| Search strategy | | |
| TITLE-ABS-KEY("student"OR"students"OR"school enrollment"OR"enrollments,school")AND  TITLE-ABS-KEY("drama education"OR"theatre education"OR"drama in education"OR"educational drama"OR"drama pedagogy"  OR"drama-based pedagogy"OR"drama-based learning"OR"process drama"OR"creative drama"OR"applied drama"OR  "drama-based instruction")AND TITLE-ABS-KEY("thinking"OR"critical thinking"OR"thinking skills"OR"communication"OR "collaboration"OR"creativity"OR"creative thinking"OR"creative ability"OR"creative abilities"OR"artistic creativity"OR  "originality"OR"innovativeness"OR"social communication"OR"interpersonal relation"OR"social relationship") | | |
| Records retrieved:179 | | |
| 5.ProQuest Central (n = 135) | | |
| Search strategy | | |
| (student OR students OR "school enrollment" OR "enrollment, school" OR "school enrollments") AND  ("drama education"OR"theatre education"OR"drama in education"OR"educational drama"OR"drama pedagogy"OR  "drama-based pedagogy"OR"process drama"OR"creative drama"OR"applied drama"OR"drama-based learning"OR  "drama-based instruction")AND("thinking"OR"critical thinking"OR"thinking skills"OR"creativity"OR"creative thinking"OR  "creative ability"OR"communication"OR"collaboration"OR"originality"OR"innovative thinking") | | |
| Records retrieved:135 | | |

Supplementary Table S2

Quality assessment of the included studies using the Joanna Briggs Institute (JBI) Critical Appraisal Checklist for Quasi-Experimental Studies

| **Author(s)** | **Study design** | **Q1** | **Q2** | **Q3** | **Q4** | **Q5** | **Q6** | **Q7** | **Q8** | **Q9** | **Score** |
| --- | --- | --- | --- | --- | --- | --- | --- | --- | --- | --- | --- |
| Elif GÖKÇEARSLAN ÇİFTÇİ et al. | CCT | Y | U | Y | Y | Y | U | Y | Y | Y | 7/9 |
| Sedighe Momeni et al. | RCT | Y | Y | Y | Y | Y | U | Y | Y | Y | 8/9 |
| Hisham Saad Zaghloul | CCT | Y | Y | Y | Y | Y | U | Y | Y | Y | 8/9 |
| Sema Karakelle | CCT | Y | Y | Y | Y | Y | U | Y | Y | Y | 8/9 |
| Graça Boal-Palheiros et al. | RCT | Y | Y | Y | Y | Y | U | Y | Y | Y | 8/9 |
| Seden Abacı et al. | RCT | Y | Y | Y | Y | Y | U | Y | Y | Y | 8/9 |
| Edanur UZUN | RCT | Y | Y | Y | Y | Y | U | Y | Y | Y | 8/9 |
| Emel ARSLAN et al. | RCT | Y | Y | Y | Y | Y | U | Y | Y | Y | 8/9 |
| Murat Ali Çınar et al. | CCT | Y | Y | Y | Y | U | U | Y | Y | Y | 7/9 |
| Fatma Betül Şenol et al. Nilgün Metin | CCT | Y | Y | Y | Y | Y | Y | Y | Y | Y | 9/9 |
| Dr. BashayerRaghianAlbalawi | RCT | Y | Y | Y | Y | Y | U | Y | Y | Y | 8/9 |
| Steven J. Holochwost et al. | CCT | Y | Y | Y | Y | Y | U | Y | Y | Y | 8/9 |
| AYÇA ENGİN GÖKBEL | RCT | Y | Y | Y | Y | Y | U | Y | Y | Y | 8/9 |
| Macarena-Paz Celume et al. | RCT | Y | Y | Y | Y | Y | U | Y | Y | Y | 8/9 |
| Maite Garaigordobil et al. | RCT | Y | Y | Y | Y | Y | U | Y | Y | Y | 8/9 |
| Sehran Dilmaç et al. | CCT | Y | Y | Y | Y | Y | U | Y | Y | Y | 8/9 |
| Münevver Can Yaşar et al. | RCT | Y | Y | Y | Y | Y | Y | Y | Y | Y | 9/9 |
| Gregory D. Freeman et al. | RCT | Y | Y | Y | Y | Y | U | Y | Y | Y | 8/9 |
| Aysun GÜNDOĞAN et al. | CCT | Y | Y | Y | Y | Y | U | Y | Y | Y | 8/9 |
| Anna Hui et al. | RCT | Y | Y | Y | Y | Y | U | Y | Y | Y | 8/9 |
| Mathieu Hainselin et al. | CCT | Y | Y | Y | Y | Y | U | Y | Y | Y | 8/9 |
| Aikaterini Dima et al. | CCT | Y | Y | Y | Y | Y | U | Y | Y | Y | 8/9 |
| Birsel Molu et al. | RCT | Y | Y | Y | Y | Y | U | Y | Y | Y | 8/9 |
| Aikaterini Dima et al. | RCT | Y | Y | Y | Y | Y | U | Y | Y | Y | 8/9 |
| Yuanyi Liu | CCT | Y | Y | Y | Y | Y | U | Y | Y | Y | 8/9 |
| Feyzullah Şahin | RCT | Y | Y | Y | Y | Y | Y | Y | Y | Y | 9/9 |
| Fatma Sac¸ li Uzunöz et al. | RCT | Y | U | Y | Y | Y | U | Y | Y | Y | 7/9 |

**Note:**

Q1. Is it clear in the study what is the cause and what is the effect?

Q2. Were the participants included in any comparisons similar?

Q3. Were the participants included in any comparisons receiving similar treatment/care other than the intervention of interest?

Q4. Was there a control group?

Q5. Were there multiple measurements of the outcome both pre and post the intervention?

Q6. Was follow-up complete and, if not, were differences between groups in terms of their follow-up adequately described and analyzed?

Q7. Were the outcomes measured in the same way for comparisons?

Q8. Were outcomes measured in a reliable way?

Q9. Was appropriate statistical analysis used?

**Abbreviations:**

JBI = Joanna Briggs Institute; RCT = randomized controlled trial; CCT = controlled clinical trial; Y = Yes; N = No; U = Unclear.

Score represents the number of JBI criteria fulfilled (maximum = 9).

**Supplementary Table S3**

Extracted post-intervention data used for the meta-analysis of creativity, critical thinking, communication, and collaboration.

| **Study** | **Outcome** | **Outcome subgroup** | **Experimental**  **Mean ± SD (n)** | **Control**  **Mean ± SD (n)** | **SMD (95% CI)** |
| --- | --- | --- | --- | --- | --- |
| Albalawi et al. (2014) | Creativity | Overall | 102.86 ± 22.73 (22) | 54.15 ± 20.45 (20) | 2.20 (1.42, 2.99) |
| Engin Gökbel et al. (2019) | Creativity | Overall | 43.12 ± 9.52 (17) | 35.39 ± 9.93 (13) | 0.78 (0.02, 1.53) |
| Garaigordobil et al. (2011) | Creativity | Overall | 58.23 ± 30.33 (53) | 26.52 ± 17.55 (33) | 1.20 (0.73, 1.67) |
| Gündoğan et al. (2013) | Creativity | 10-year-old children | 1.62 ± 0.20 (30) | 1.38 ± 0.42 (30) | 0.72 (0.20, 1.24) |
| Gündoğan et al. (2013) | Creativity | 13-year-old children | 1.50 ± 0.25 (30) | 1.33 ± 0.25 (30) | 0.67 (0.15, 1.19) |
| Hainselin et al. (2018) | Creativity | Overall | 1.514 ± 0.426 (18) | 0.812 ± 0.368 (12) | 1.69 (0.83, 2.55) |
| Karakelle (2009) | Creativity | Overall | 40.93 ± 14.90 (15) | 27.40 ± 9.94 (15) | 1.04 (0.27, 1.81) |
| Liu (2023) | Creativity | Overall | 72.45 ± 5.85 (30) | 66.88 ± 4.91 (30) | 1.02 (0.48, 1.56) |
| Molu et al. (2024) | Creativity | Overall | 98.09 ± 8.23 (33) | 95.27 ± 13.13 (33) | 0.25 (-0.23, 0.74) |
| Momeni et al. (2017) | Creativity | 4-year-old children | 38.00 ± 8.27 (5) | 33.00 ± 1.41 (2) | 0.57 (-1.13, 2.27) |
| Momeni et al. (2017) | Creativity | 5-year-old children | 40.00 ± 6.38 (12) | 38.57 ± 8.30 (7) | 0.19 (-0.74, 1.13) |
| Momeni et al. (2017) | Creativity | 6-year-old children | 50.75 ± 6.95 (16) | 46.20 ± 6.08 (10) | 0.66 (-0.15, 1.48) |
| Şahin (2022) | Creativity | Fluency | 10.31 ± 4.73 (16) | 6.47 ± 1.81 (15) | 1.03 (0.28, 1.79) |
| Yaşar et al. (2012) | Creativity | Overall | 64.30 ± 7.40 (40) | 31.00 ± 10.70 (40) | 3.58 (2.87, 4.30) |
| Çınar et al. (2019) | Critical Thinking | Overall | 196.10 ± 22.11 (40) | 200.44 ± 19.17 (35) | −0.21 (−0.66, 0.25) |
| Dilmaç et al. (2020) | Critical Thinking | Overall | 62.00 ± 5.10 (25) | 59.60 ± 9.57 (25) | 0.31 (−0.25, 0.87) |
| Dima & Tsiaras (2020) | Critical Thinking | Primary school study (n=25) | 2.64 ± 1.186 (25) | 2.28 ± 1.06 (25) | 0.32 (−0.24, 0.87) |
| Dima, Kaiafa & Tsiaras (2020) | Critical Thinking | Primary school study (n=200) | 2.64 ± 1.19 (200) | 2.28 ± 2.28 (200) | 0.20 (0.00, 0.39) |
| Saçlı Uzunöz et al. (2017) | Critical Thinking | Overall | 283.95 ± 35.318 (34) | 260.44 ± 35.318 (30) | 0.66 (0.15, 1.16) |
| Uzun (2019) | Critical Thinking | Overall | 62.00 ± 5.32 (25) | 59.60 ± 9.36 (25) | 0.31 (−0.25, 0.87) |
| Zaghloul (2018) | Critical Thinking | Male students | 46.63 ± 1.73 (35) | 23.17 ± 2.38 (35) | 11.13 (9.18, 13.09) |
| Zaghloul (2018) | Critical Thinking | Female students | 22.97 ± 2.61 (35) | 24.86 ± 2.28 (35) | −0.76 (−1.25, −0.28) |
| Emel Arslan et al. (2010) | Communication | Overall | 74.00 ± 6.84 (24) | 63.42 ± 6.98 (24) | 1.51 (0.86, 2.15) |
| Graça Boal-Palheiros et al. (2023) | Communication | Overall | 23.27 ± 4.08 (56) | 23.21 ± 4.14 (52) | 0.01 (−0.36, 0.39) |
| Murat Ali Çınar et al. (2019) | Communication | Overall | 104.23 ± 11.28 (40) | 105.92 ± 10.85 (35) | −0.15 (−0.61, 0.30) |
| Elif Gökçearslan Çiftçi et al. (2017) | Communication | Overall | 65.00 ± 5.30 (28) | 61.20 ± 4.50 (26) | 0.76 (0.21, 1.31) |
| Anna Hui et al. (2006) | Communication | Overall | 2.60 ± 0.71 (126) | 2.17 ± 0.69 (69) | 0.61 (0.31, 0.91) |
| Hisham Saad Zaghloul (2018) | Communication | Male students | 46.80 ± 1.997 (35) | 23.43 ± 2.477 (35) | 10.27 (8.46, 12.09) |
| Hisham Saad Zaghloul (2018) | Communication | Female students | 46.80 ± 1.891 (35) | 22.97 ± 2.515 (35) | 10.59 (8.73, 12.46) |
| Seden Abacı et al. (2015) | Collaboration | Overall | 22.47 ± 2.17 (15) | 17.93 ± 3.92 (15) | 1.39 (0.58, 2.20) |
| Graça Boal-Palheiros et al. (2023) | Collaboration | Overall | 23.21 ± 4.25 (56) | 24.90 ± 4.22 (52) | −0.40 (−0.78, −0.01) |
| Macarena-Paz Celume et al. (2020) | Collaboration | Overall | 4.50 ± 0.30 (61) | 3.50 ± 0.30 (65) | 3.31 (2.77, 3.86) |
| Gregory D. Freeman et al. (2003) | Collaboration | Overall | 100.76 ± 17.39 (49) | 105.70 ± 19.00 (44) | −0.27 (−0.68, 0.14) |
| Steven J. Holochwost et al. (2024) | Collaboration | Overall | 3.27 ± 1.17 (46) | 2.68 ± 0.95 (47) | 0.55 (0.14, 0.96) |
| Fatma Betül Şenol & Nilgün Metin (2021) | Collaboration | Overall | 28.67 ± 0.91 (18) | 15.39 ± 1.33 (18) | 11.40 (8.53, 14.26) |

**Note:** Values are presented as post-intervention means, standard deviations (SDs), and sample sizes (n) extracted from the original studies. Standardized mean differences (SMDs) were calculated using Review Manager (RevMan) version 5.4 based on post-intervention data. Positive SMD values indicate greater improvement in the educational drama intervention group compared with the control group.
